# Differential transcriptomic host responses in the early phase of viral and bacterial infections in human lung tissue explants ex vivo

**DOI:** 10.1186/s12931-024-02988-8

**Published:** 2024-10-12

**Authors:** Aaqib Sohail, Fakhar H. Waqas, Peter Braubach, Laurien Czichon, Mohamed Samir, Azeem Iqbal, Leonardo de Araujo, Stephan Pleschka, Michael Steinert, Robert Geffers, Frank Pessler

**Affiliations:** 1 Research Group Biomarkers for Infectious Diseases, TWINCORE Centre for Experimental and Clinical Infection Research—a joint venture of Hannover Medical School and the Helmholtz Centre for Infection Research, Hannover, Germany; 2 Institute for Pathology, Hannover Medical School, Hannover, Germany; 3 Department of Zoonoses, Faculty of Veterinary Medicine, Zagazig University, Zagazig, Egypt; 4 Institute of Medical Virology, Justus-Liebig-Universität, 35390 Giessen, Germany; 5 German Center for Infection Research (DZIF), Partner Site Giessen, Giessen, Germany; 6 Institute for Microbiology, Technical University Braunschweig, Brunswick, Germany; 7 Genome Analysis, Helmholtz Centre for Infection Research, Brunswick, Germany; 8 Centre for Individualised Infection Medicine, Hannover, Germany; 9 Research Group Biomarkers for Infectious Diseases, Helmholtz Centre for Infection Research, Brunswick, Germany; 10 Present Address: Molecular and Experimental Mycobacteriology Group, Research Center Borstel, Leibniz Lung Center, Borstel, Germany

**Keywords:** Biomarker, Chronic obstructive pulmonary disease, Emphysema, Gene expression, Lung tissue, Infection, Long noncoding RNA, Pneumonia, miRNA, PIWI-associated RNA, Prokineticin 2, Transcription

## Abstract

**Background:**

The first 24 h of infection represent a critical time window in interactions between pathogens and host tissue. However, it is not possible to study such early events in human lung during natural infection due to lack of clinical access to tissue this early in infection. We, therefore, applied RNA sequencing to ex vivo cultured human lung tissue explants (HLTE) from patients with emphysema to study global changes in small noncoding RNA, mRNA, and long noncoding RNA (lncRNA, lincRNA) populations during the first 24 h of infection with influenza A virus (IAV), *Mycobacterium bovis* Bacille Calmette-Guerin (BCG), and *Pseudomonas aeruginosa*.

**Results:**

*Pseudomonas aeruginosa* caused the strongest expression changes and was the only pathogen that notably affected expression of microRNA and PIWI-associated RNA. The major classes of long RNAs (> 100 nt) were represented similarly among the RNAs that were differentially expressed upon infection with the three pathogens (mRNA 77–82%; lncRNA 15–17%; pseudogenes 4–5%), but *lnc-DDX60-1*, *RP11-202G18.1*, and *lnc-THOC3-2* were part of an RNA signature (additionally containing *SNX10* and *SLC8A1*) specifically associated with IAV infection. IAV infection induced brisk interferon responses, *CCL8* being the most strongly upregulated mRNA. Single-cell RNA sequencing identified airway epithelial cells and macrophages as the predominant IAV host cells, but inflammatory responses were also detected in cell types expressing few or no IAV transcripts. Combined analysis of bulk and single-cell RNAseq data identified a set of 6 mRNAs (*IFI6*, *IFI44L*, *IRF7*, *ISG15, MX1*, *MX2*) as the core transcriptomic response to IAV infection. The two bacterial pathogens induced qualitatively very similar changes in mRNA expression and predicted signaling pathways, but the magnitude of change was greater in *P. aeruginosa* infection. Upregulation of *GJB2*, *VNN1*, *DUSP4*, *SerpinB7*, and *IL10*, and downregulation of *PKMYT1*, *S100A4*, *GGTA1P*, and *SLC22A31* were most strongly associated with bacterial infection.

**Conclusions:**

Human lung tissue mounted substantially different transcriptomic responses to infection by IAV than by BCG and *P. aeruginosa*, whereas responses to these two divergent bacterial pathogens were surprisingly similar. This HLTE model should prove useful for RNA-directed pathogenesis research and tissue biomarker discovery during the early phase of infections, both at the tissue and single-cell level.

**Supplementary Information:**

The online version contains supplementary material available at 10.1186/s12931-024-02988-8.

## Introduction

The first 24 h of infection form a critical time window in interactions between many pathogens and host tissue, as they include attachment to and invasion of target cells, recognition of pathogens by cellular sensors and, in many cases, the first replication cycles of the pathogen. Insights into changes in host RNA expression are particularly important for our understanding of this early phase because they mediate a major part of the immediate early cell and tissue responses by enabling the required coordinated changes in gene expression. In addition, due to the high dynamic range in expression change of long RNAs and the association of small RNAs such as microRNA (miRNA) with central regulatory switches, RNA molecules have proven high potential as accurate biomarkers for disease-specific processes. For pulmonary infections, this has predominantly been shown by analysis of peripheral blood RNA from patients with established disease (e.g., [1–3]). However, to better capture pathophysiological processes in the target organ itself, it would be highly desirable to analyze lung tissue directly. While this is technically possible by transbronchial biopsy and may be clinically justified in difficult-to-diagnose cases, it is usually not clinically or ethically feasible within the very early phase of infection with a known causative pathogen. Even though there are well-established animal and cell culture models to study human lower respiratory tract infections, they cannot fully represent the events seen in intact human lung tissue. Organotypic culture of human lung tissue explants (HLTE) has been successfully used to overcome some of these drawbacks. The HLTE model is based on ex vivo cultivation of human lung tissue that has been removed for medical reasons, for instance in the context of tumor surgery or lung transplantation. The majority of studies have featured a refined version of the HLTE model based on precision cut lung slices (PCLS, reviewed in refs. [4, 5]). In this model, the tissue is embedded in low-melting agarose and then sectioned, thus preserving tissue architecture better during subsequent culture. However, the PCLS model is labor-intensive and embedding the tissue in agarose may interfere with subsequent analyses of the tissue, for instance by RNA sequencing. The classic organotypic HLTE model, therefore, still constitutes an attractive option, in particular if more sophisticated tissue analyses by –omics technologies are planned. Regarding respiratory infections, it has been demonstrated that HLTEs can support invasion and replication of viral, bacterial, and fungal pathogens and reproduce cardinal aspects of the expected host responses [6–17]. Tumor-free tissue margins from lung tumor surgery were used in most of these studies, but tissue from patients with chronic obstructive pulmonary disease (COPD) was successfully used to study cytokine responses to *H. influenza* and influenza A virus (IAV) [14, 17, 18] and the anti-inflammatory effect of citraconic acid on IAV infection [19]. Explanted lung tissue from patients with an indication for lung transplantation for lung failure from obstructive lung disease would, therefore, be an attractive source of HLTEs for studies of lower respiratory infections in medical centers with a high volume of lung transplantation. However, it remains unknown whether tissue which has been compromised by a long-standing disease process leading to respiratory insufficiency not only supports replication of key human pathogens ex vivo but also yields valid data about transcriptomic host responses.

RNA sequencing enables profiling of both long and small RNAs of different classes from a single sample, which is referred to as integrative RNA sequencing. For infection research, a particularly attractive feature is that expression of viral RNAs is also captured from the same sample, allowing to identify infected host cells and to compare host responses in infected vs. noninfected (bystander) cells. Both bulk and single-cell RNA sequencing have been employed to characterize RNA expression changes in human lung tissue. For instance, Alfi et al. applied bulk RNA sequencing to HLTEs from tumor surgery to assess transcriptomic responses to SARS-CoV-2 in upper and lower respiratory tract [16], whereas single-cell RNA sequencing (scRNAseq) of PCLS was recently used to study antiviral drugs against SARS-CoV-2 [20]. However, there are no studies featuring bulk or scRNAseq of organotypic HLTEs to compare transcriptomic tissue responses to viral vs. bacterial pathogens.

The aims of the present study were (i) to describe differences in early transcriptomic host responses due to viral vs. bacterial infections of human lung tissue ex vivo, (ii) to dissect host responses to IAV at the single-cell level, and (iii) to identify IAV infected cell types. Specifically, we have adapted HLTE culture of tissue from patients with indication for lung transplantation due to advanced emphysema and used (i) integrated RNA sequencing of small and long RNA classes to evaluate early RNA expression changes following infection with IAV, *Mycobacterium bovis* Bacille Calmette-Guerin (BCG), and *Pseudomonas aeruginosa*, and (ii) single-cell RNA sequencing to identify host mRNA expression changes and expression of viral transcripts in IAV infection at the single cell level. We find that the tissue explants faithfully reproduce the expected pathogen-specific responses at the tissue and single-cell level and allow (i) validating, in human tissue, RNA biomarkers that had been previously identified in cellular or animal models, and (ii) the discovery of novel infection-associated RNA molecules that can be validated in future studies.

## Results

**Fig. 1:**
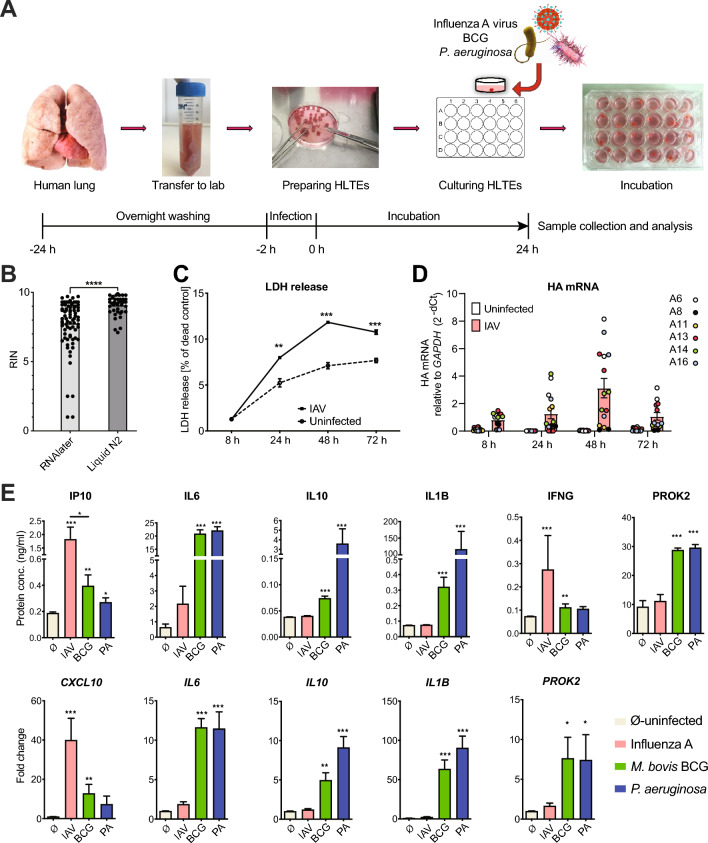
Experimental design and functional validation of the human lung tissue explants (HLTE) model. **A** Outline of the experimental procedure. After removal from the explanted lung, tissue was divided into pieces of approx. 30 mg and then incubated in medium overnight. HLTE pieces were infected with 2.0 × 10^5^ FFU/ml of influenza virus strain A/Giessen /6/2009 H1N1 (in short IAV), 5 × 10^6^ CFU/ml *M. bovis* strain H37Rv (BCG), or 1 × 10^8^/ml *P. aeruginosa* strain PA14. All analyses were performed 24 h post infection (p.i.) unless indicated otherwise. **B** Comparison of two methods of tissue preservation before RNA extraction. HLTE pieces were either snap frozen in liquid nitrogen (*n* = 20) or incubated in RNAlater at 4 °C overnight (n = 38) before storage at − 80 °C, followed by RNA extraction. Y-axis = RNA integrity number (RIN). **C** Time course of LDH release from uninfected or IAV-infected HLTE pieces (*n* = 3 per group). LDH was measured in tissue culture supernatants and is expressed as % of LDH extracted from lysed tissue control. **D** Time course of IAV hemagglutinin (HA) mRNA levels (RT-qPCR), indicating transcription of viral RNA (*n* = 6). **E** Differences in cytokine/chemokine induction by infection with IAV, BCG, and *P. aeruginosa*. Upper row: protein concentrations measured by EIA. Bottom row: mRNA levels measured by RT-qPCR relative to mock-infected HLTE, using GAPDH as internal reference. *n* = 9. *p ≤ 0.05; **p ≤ 0.01; ***p ≤ 0.001; ****p ≤ 0.0001; **B**–**D** = unpaired parametric T test; **E** = Mann–Whitney U test. Data represent means ± SEM

*Establishment of the infection model*. The work flow of a typical experiment is shown in Fig. 1A. In order to optimize RNA quality for RNAseq, we compared two methods for RNA isolation. In the first method, the lung tissue pieces were incubated with RNAlater for 24 h at 4 °C and then stored at − 80 °C in RNAlater prior to extraction. In the second method, they were snap frozen in liquid nitrogen and then stored at − 80 °C. The liquid nitrogen method yielded significantly higher RNA Integrity Numbers (Fig. 1B) and was thus used throughout. We measured LDH release in uninfected and IAV-infected HLTEs in order to assess spontaneous degradation of tissue during culturing and potential virus-induced cytopathic effects. LDH was released throughout the 72 h time course, reaching maximum values of about 7% and 12% in uninfected and infected HLTEs, respectively, indicating a mild-moderate cytopathic effect (Fig. 1C). To assess whether the HLTE model supports release of active IAV particles, we employed the immuno-foci assay to quantify replication-competent virus particles in the supernatant and measured viral RNA transcription through HA mRNA quantification. An apparent decline of replication-competent virus particles in the supernatant was noted over a 72 h period (Figure S1A). This was likely due to the fact that the initial viral inoculum was not replaced by virus-free medium, and it was thus concluded that viral titers in supernatant could not be used to measure viral replication. On the other hand, viral HA mRNA expression increased steadily and peaked by 48 h post infection (p.i.) (Fig. 1D). However, significant inter- and intra-donor variation was observed, likely due to differences in tissue integrity and cell type proportions between samples. To verify whether the tissues could mount the expected differential responses to IAV and the two bacterial pathogens (*P. aeruginosa* and BCG) in the first 24 h of infection, we measured several classic cytokines in supernatants and the corresponding mRNAs in tissue lysates (Fig. 1E). As expected, CXCL10 and IFNα levels were highest in influenza infection, whereas IL6, IL10, IL1β, and PROK2 (which is highly inducible in macrophages by LPS and other TLR2/4 agonists [21]) reached higher levels in the bacterial infections. Taken together, these results suggested that HLTEs from emphysema patients constituted a valid model to study early transcriptomic tissue responses to viral and bacterial infections ex vivo.

*Bulk RNA sequencing*. Using HLTEs from five separate donors, we performed 5 independent infection experiments, each featuring infection with all three pathogens in parallel. We then applied RNAseq to measure expression of sncRNA, long noncoding, pseudogene, and protein-coding RNA in uninfected HLTEs and in HLTEs infected with IAV, BCG, or *P. aeruginosa*. To reduce the impact of intra-donor variability observed in the IAV infection time course shown in Fig. 1D, we pooled RNA from 2 to 4 HLTE pieces from each donor so that five pooled RNA samples per condition were available for RNAseq (final *n* = 20).

**Fig. 2:**
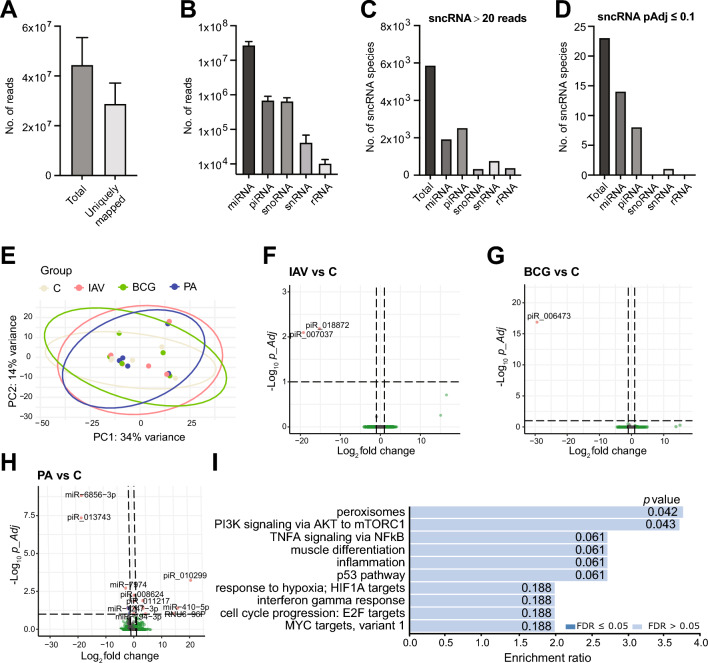
Modest reprogramming in sncRNA expression in HLTE in viral and bacterial infections. sncRNA populations were determined by sncRNAseq 24 h after infection with IAV, BCG, and *P. aeruginosa*, or after mock infection (n = 5 per condition). Results of the DE analysis with DEseq2 are found in Table S1. **A** Detection efficiency (no. of reads) of all sncRNAs vs. sncRNAs mapping to human genome hg38. **B** Abundance of the major sncRNA subpopulations miRNA, piRNA, snoRNA, and snRNA. **C** Abundance of sncRNA subtypes detected at a total of ≥ 20 reads in all samples. **D** Abundance of differentially expressed sncRNA subtypes (pAdj. ≤ 0.1). **E** PCA showing poor separation of the four groups based on sncRNA. **F**–**H** Volcano plots showing DE sncRNAs (pAdj ≤ 0.1, fold change [FC], ≥|2|). **F** IAV vs. uninfected tissue. **G** BCG vs. uninfected tissue. **H** *P. aeruginosa* vs. uninfected tissue. **I**, Hallmark GSEA analysis of predicted targets of DE miRNA in *P. aeruginosa* infection

*Modest reprogramming of small noncoding RNA (sncRNA) populations*. Small (< 50 nucleotides long) RNA sequencing of total RNA extracted 24 h p.i. revealed the expected sequencing depth: a mean 8.3*10^7^ reads were obtained per sample; a mean 4.4*10^7^ of these passed the length filter, of which 2.8*10^7^ could be mapped to the human genome (hg38) (Fig. 2A). By sncRNA class, most reads mapped to miRNAs and substantially fewer to PIWI-interacting RNA (piRNA) and small nucleolar RNA (snoRNA) (Fig. 2B). Very few reads mapped to small nuclear RNA (snRNA) and ribosomal RNA (rRNA), which was consistent with the high quality of the input RNA. After filtering out lowly expressed sncRNA (< 20 reads in the four experimental groups combined), piRNA contributed the highest number of sncRNA species, followed by miRNA. However, the majority of the differentially expressed (DE; *p*-Adj ≤ 0.1) sncRNA species were miRNA followed by piRNA (Fig. 2C,D). A principal component analysis (PCA) did not reveal global differences in the sncRNA populations of the 4 groups (Fig. 2E). Indeed, there were only 2 DE piRNAs in IAV infection vs. uninfected tissue and 1 DE piRNA in BCG infection vs. uninfected tissue, and their expression in uninfected tissue was already low, indicating that their functional significance is uncertain (Fig. 2F,G**)**. On the other hand, more substantial expression changes were observed in *P. aeruginosa* infection. 20 sncRNA were DE: 13 miRNA, 6 piRNA, and 1 snRNA (Fig. 2H). Among the DE miRNAs, mir-410-5p and miR-665 have been linked to increased cell proliferation and are candidate biomarkers for cancer. Decreased expression of one of the downregulated miRNA, miR-7974, has been reported in hypoxia [22] and oxidative stress [23], and increased expression in breast tumor tissue [24]. A gene set enrichment (GSEA) analysis of predicted mRNA targets of the miRNA DE in *P. aeruginosa* infection suggested activation of pathways related to peroxisomes, inflammation, and cell cycle (Fig. 2I). However, statistical significance was modest. Nonetheless, DE of about 10% of the predicted mRNA targets could be verified in the long RNAseq dataset (Figure S1B) and DE of additional predicted targets would be expected at later time points. piRNAs were the 2nd most significantly DE sncRNA class in *P. aeruginosa* infection and were notably overrepresented among the most strongly DE sncRNA (e.g., piR_010299). piRNAs have been implicated in tissue responses to infections in humans [3] and animals [25], suggesting that their DE in this HLTE model is pathophysiologically significant.

**Fig. 3:**
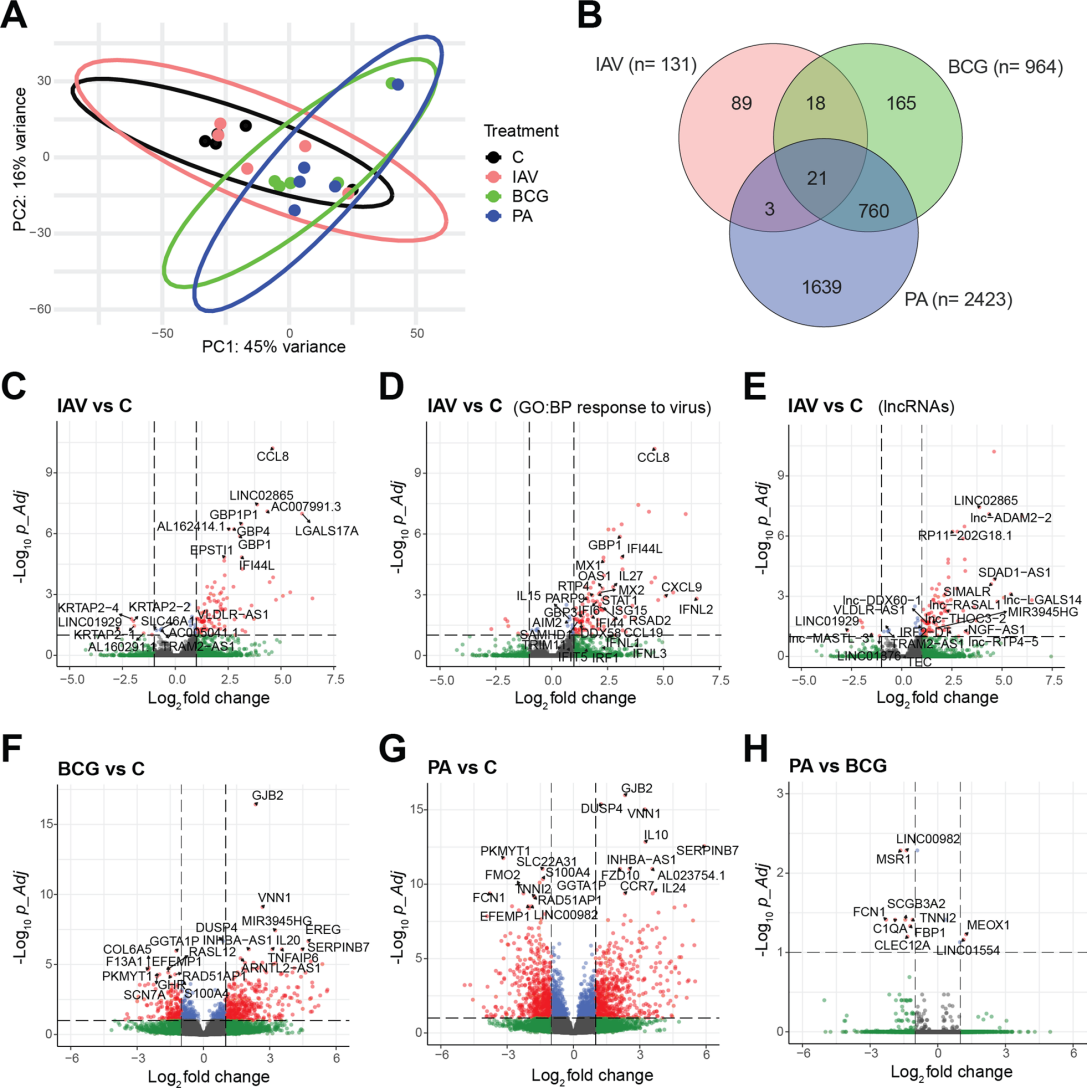
Differential reprogramming of RNA populations by viral and bacterial infection. Using RNA from the same total RNA samples as used for small RNA sequencing (Fig. 2), long RNA populations were determined by RNAseq 24 h after infection or mock treatment (n = 5 per condition). **A** PCA indicating somewhat better separation than with sncRNAseq (compare Fig. 2E). **B** Venn diagram showing the number of DE mRNAs (pAdj ≤ 0.1, FC ≥|2| with respect to uninfected tissue) unique to each pathogen, shared between any two pathogens, or common to all three. **C**–**H** Volcano plots identifying DE mRNA and lncRNA species with respect to uninfected tissue in infection with IAV (**C**–**E**), BCG (**F**), and *P. aeruginosa* (**G**), and comparing *P. aeruginosa* vs. BCG infected HLTEs (**H**). Cutoffs were set at FC =|2| (i.e. log_2_ =|1|) and *p*-Adj 0.1 (i.e. − log_10_ = 1)

*Marked reprogramming in long RNA (mRNA, long noncoding RNA, pseudogene RNA) populations reveals signatures for bacterial vs. viral infection*. Sequencing of long RNA (> 100 nucleotides long) revealed deep coverage in all samples in that we obtained a mean (SD) 42.1 × 10^6^ (± 7.3 × 10^6^) reads mapping to protein-coding RNA (mRNA), long noncoding RNA (lncRNA and long intervening noncoding RNA, lincRNA), and pseudogene RNA species. To examine the spread of RNAseq data across the four groups, we generated a graphical representation of Fragments Per Kilobase Million (FPM) for each group. The plot illustrates that the data are equally distributed across all groups (Figure S2A). With respect to both total and DE RNA, the majority of reads mapped to protein-coding RNA, followed by lncRNA and then pseudogene RNA in infection with all three pathogens (Figure S2B–D), whereby the percentages of the four captured RNA classes (protein-coding RNA, lncRNA, lincRNA, pseudogenes) in the DE RNAs were similar across infection with the three pathogens (Figure S2E). A PCA based on all species of long RNA suggested similarities between IAV infection and control samples on the one hand and between the two bacterial infections on the other hand (Fig. 3A). Still, no clear separation was seen even though expression changes were substantially greater than in the sncRNA populations. Nonetheless, it was evident that the extent of differential expression increased from IAV (n = 131 DE RNAs) over BCG (n = 964 DE RNAs) to *P. aeruginosa* (n = 2423 DE RNAs) (Fig. 3B).

High levels of mRNA corresponding to all 8 IAV genome segments were detected in the IAV-infected samples, but only background signals in the other three groups, thus evidencing successful IAV infection of the HLTEs used for the RNAseq analysis (Figure S3). Volcano plots were then used to visualize differences between the three pathogens in DE with respect to uninfected tissue (Fig. 3C–H). In IAV infection, 131 RNAs were DE with respect to uninfected tissue. Several mRNAs were highly upregulated which are known to be strongly associated with host responses to influenza virus infection (Fig. 3C). In particular, *CCL8*, *CXCL9*, *GBP1*, *IFI44L*, *IFI6*, *IFNL2*, *IL27*, *ISG15*, *MX1*, and *MX2* are all contained in the Gene Ontology (GO) term *BP 0009615 response to virus*** (**Fig. 3D**)**, thus providing further evidence that the HLTE mounted the expected anti-IAV innate immune response. Furthermore, we noted 19 lncRNAs and lincRNAs among the DE RNAs (Fig. 3E). Of note, lncRNAs *ADAM2-2* (ENSG00000253939), *SIMALR* (suppressor of inflammatory macrophage apoptosis lncRNA, ENSG00000226004), *LINC02865* (ENSG00000229922), *LGALS17A* (ENSG00000226025), and *LGALS14* (ENSG00000269460) were among the most highly upregulated transcripts in IAV infection (Fig. 3E). *lnc-ADAM2-2* is a novel gene which encodes antisense RNA to the genes encoding the IDO1 and IDO2 enzymes, both of which have immunomodulatory functions in influenza virus infection [26, 27]. lncRNA *SIMALR* and lincRNA *LINC02865* (a suppressor of inflammatory macrophage apoptosis) are transcribed from overlapping sequences on chromosome 6. They have emerged as novel regulators of macrophage biology by suppressing inflammatory macrophage apoptosis via Netrin-1 (NTN1) [28] and are located just downstream of the gene encoding the anti-inflammatory deubiquitinase TNFAIP3 (also known as A20). *LGALS17A* and its downstream lncRNA *lnc-LGALS14* share a genomic location on chromosome 19 with galectins, which possess antiviral functionality in IAV infection [29]. *LGALS17A* and *lnc-LGALS14* are derived from a galectin pseudogene, and *LGALS17A* has previously been identified as an IFN-stimulated transcript [30]. In addition, they are located downstream of a gene cluster relating to type III IFN responses, of which INFL2 was significantly upregulated in the current dataset, whereas upregulation of IFNL1 and 3 was less significant (*p* Adj > 0.1). We then aimed to validate the expression of a subset of the DE lncRNA, lincRNA and pseudogene RNAs by RT-qPCR. Robust PCR assays could be established for *RP11-202G18.1*, *GBP1P1*, *SIMALR*, *AC093063.1*, and *LGALS17A.* Indeed, expression of all five increased significantly 24 h after IAV infection, but to lower levels than *CXCL10* mRNA, which was measured for comparison (Figure S4).

Transcriptomic tissue responses to the two bacterial pathogens were much more extensive than to IAV (Fig. 3F,G), and there was considerable overlap in DE RNAs, even though *P. aeruginosa* and BCG belong to two different phyla and differ significantly in their interactions with host cells. 760 long RNA species were DE (with respect to uninfected tissue) in infection by both bacterial pathogens, but not in IAV infection (Fig. 3B), and a four-way plot based on Wald statistic suggested that all genes that were DE by both bacterial pathogens were regulated in the same direction (Figure S5). This plot identified *GJB2* as the gene that was most robustly upregulated in both bacterial infections. *GJB2* encodes gap junction binding protein 2, a connexin which plays roles in epithelial barrier integrity [30] and is known for its importance in lung adenocarcinoma. Little is known about its role in inflammation or infections, but it is listed in GO:BP gene set RESPONSE_TO_BACTERIUM (GO:0009617). *VNN1* encodes vanin 1, an oxidative stress sensor that is part of the tissue response to oxidative stress and inflammation in a variety of organs [31]. The most specifically and strongly regulated RNAs were found in *P. aeruginosa* infection. In addition to *GJB2* and *VNN1*, the most significantly (Wald test statistic) upregulated mRNAs were *DUSP4* (encoding dual specificity phosphatase 4, a protein tyrosine/serine/threonine phosphatase involved in mitogenic signal transduction), *IL10* (encoding a predominantly anti-inflammatory cytokine [32]), and *SERPINB7* (encoding a member of the serpin family of protease inhibitors, which have anti-inflammatory and anticoagulant properties and counteract proteolytic tissue destruction [33]). The RNAs that were most significantly downregulated by both pathogens were *PKMYT1* (encoding a kinase that inhibits G2/M transition in the cell cycle [34]), *GGTA1P* (encoding an enzymatically inactive alpha-1,3-galactosyltransferase involved in immune regulation (e.g., [35]), and *S100A4* (encoding the S100 Ca^++^-binding protein S4, which furthers cell motility, migration, and invasion [36]). All three genes were downregulated more significantly by *P. aeruginosa* than by BCG. In spite of the strong agreement between *P. aeruginosa* and BCG in terms of directionality of DE, some degree of DE between the two bacterial infections was apparent. This is evidenced in the four-way plot (see RNAs marked in green [significant only in BCG infection] and violet [significant only *in P. aeruginosa* infection] in Figure S5 as well as in the volcano plot shown in Fig. 3H**.**

**Fig. 4:**
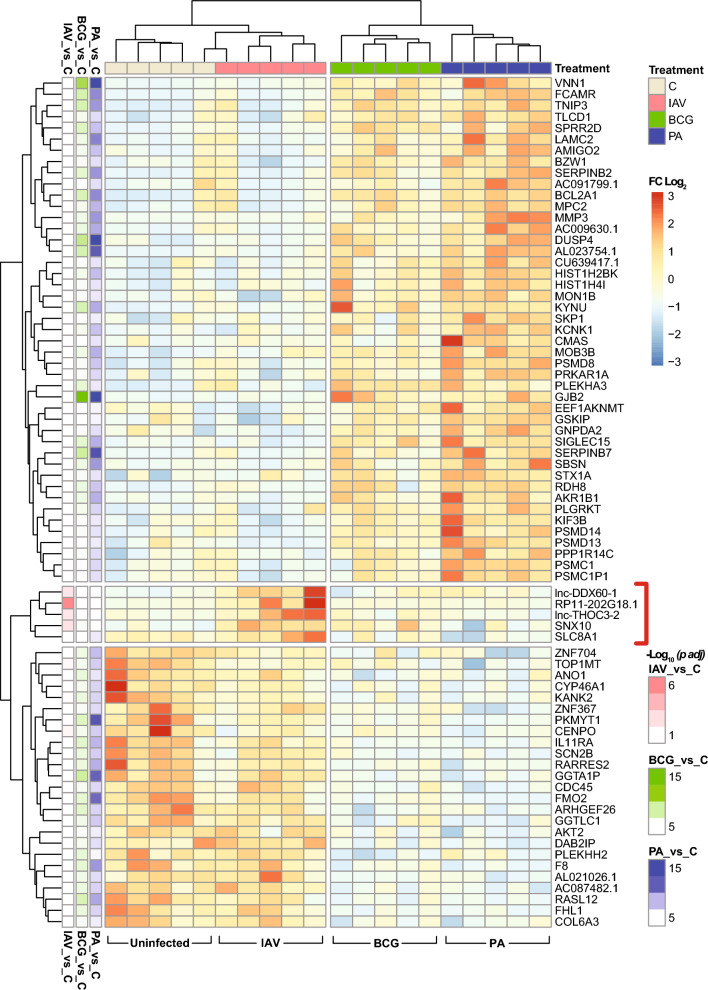
Viral and bacterial infections leave different RNA signatures in HLTEs. Unsupervised hierarchical biclustering analysis based on the 75 most significantly up- or downregulated mRNA and lncRNA (*p* < 7.8E−04, ANOVA across all four groups). Red bracket: RNAs comprising a 5-transcript “viral signature”. The color scheme in the 3 columns on the left shows *p*-Adj values for DE due to infection with each pathogen compared with uninfected tissue

In hierarchical clustering analysis based on the 75 most significantly DE RNA selected by ANOVA across all four experimental groups (*p* values: 3.6E−06 to 7.8E−04), there was a remarkably clear separation in that the bacterial infections clustered in one clade and within it BCG and *P. aeruginosa* into separate subclades (Fig. 4). Separation of IAV infection from uninfected tissue was also excellent in that only one IAV-infected and one control sample were classified in the same subclade. Even though there were much fewer DE RNAs in IAV infection than in the bacterial infections, this hierarchical clustering analysis revealed a distinct “viral” signature, which consisted of 5 upregulated RNAs (marked red in Fig. 4). Three of these (*lnc-DDX60-1* [ENSG00000248601,] *lnc-THOC3-2* [ENSG00000248596)], and *RP11-202G18.1* [ENSG00000227531] are transcripts of unknown function which may arise during transcription of neighboring genes related to cellular responses to infection and inflammation. *SLC8A1* encodes the Na^+^/Ca^++^ exchange channel NCX1, which is expressed in a variety of cell types and plays roles in mediating airway smooth muscle responses to inflammation [37] and thrombin-induced increased endothelial permeability [38]. *SNX10* (sorting nexin 10) is an important differentiation factor of myeloid cells and supports a proinflammatory phenotype of macrophages [39].

**Fig. 5:**
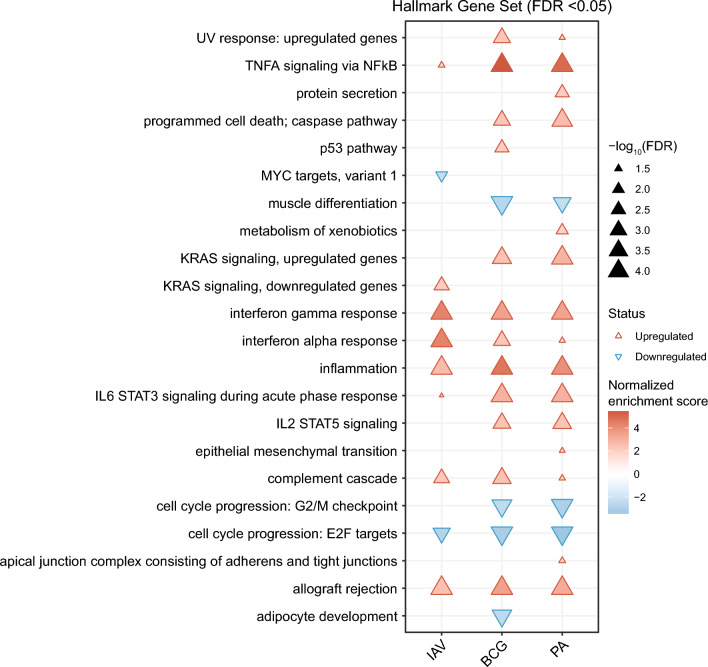
Gene set enrichment analysis (GSEA) based on Hallmark pathways identifies common and distinct signaling pathways in bacterial and viral infection of explanted human lung tissue. mRNA expression data were extracted from the bulk RNAseq data set used for Fig. 3, and a GSEA was performed using the prerank gene list from Deseq2 analysis against the Hallmark gene set collection. Significantly enriched or depleted Hallmark pathways are indicated by the triangles as detailed in the legend next to the figure. A GSEA based on Gene Ontology Biological Process (GO:BP) is shown in Figure S6

GSEA analysis based on Hallmark pathways revealed pathogen-specific regulation of functional pathways, but also common features shared among the three types of infection (Fig. 5). Several pathways were commonly upregulated by all three pathogens. These related to innate immune system signaling (*IFNG response*, *IFNA response*, *inflammatory response*, *IL6_JAK_Stat3 signaling*, *TNFA signaling *via* NFKB*), other aspects of innate immune activation (*allograft rejection*, *complement activation*), and apoptosis. One commonly downregulated pathway was *cell cycle progression: E2F targets*, indicating inhibition of cell proliferation by all three pathogens. The two bacterial infections could be distinguished from IAV infection by weaker induction of type I and type II IFN signaling, but stronger enrichment of signaling pathways such as *IL2_Stat5_signaling*, *IL6_STAT3 signaling*, *programmed cell death*, and *KRAS signaling (upregulated genes)*. In contrast, depletion of *myc targets*, reflecting decreased cellular processes and cell cycle, and enrichment of *KRAS signaling (downregulated genes)* were unique for IAV infection. The pathways uniquely regulated in *P. aeruginosa* infection reflected a more vigorous induction of compensatory tissue responses such as *formation of tight junctions, protein secretion*, and *metabolism of xenobiotics*. *Adipocyte development* and *p53 pathway* were the only two pathways that were uniquely differentially regulated in BCG-infected tissue only. Taken together, these results provide further evidence that this HLTE model recapitulates classic tissue responses to infectious agents and reveals pathophysiologically plausible differences between the responses to viral vs. bacterial pathogens.

**Fig. 6:**
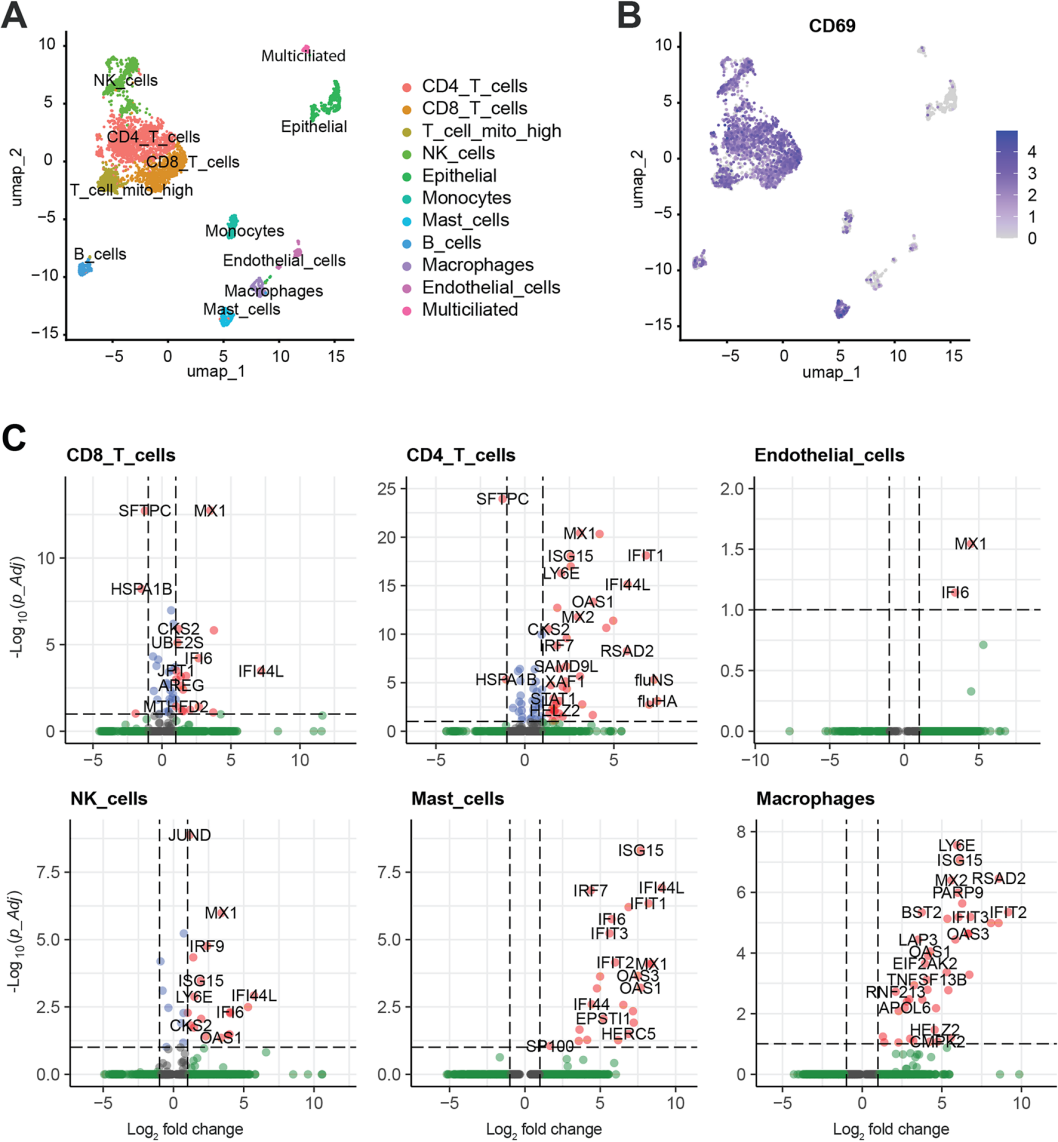
Single-cell RNA sequencing identifies strongest induction of antiviral host responses in CD4, CD8 + T, NK cells, mast cells, and macrophages. Single cell transcriptomes were determined in pooled single cell suspensions derived from HLTEs from one donor with emphysema due to COPD 24 h after IAV infection or mock infection. **A** Identification of 11 cell subpopulations by uniform manifold approximation and projection (UMAP). **B** CD69 mRNA expression (see Figure S7 for CD103 expression). **C** Volcano plots identifying DE genes in the major immune cell types and vascular ECs. The dotted lines identify cutoffs FC =|2| (i.e. log_2_ =|1|) and *p*-Adj 0.1 (i.e. − log_10_ = 1). DE in the other cell types is shown in Figure S9

*Cell type-specific responses to IAV infection*. We then tested whether the HLTE could be used to dissect interactions between IAV and lung tissue at the single-cell level and applied the 10 × genomics single-cell RNA sequencing (scRNAseq) platform to single-cell suspensions from IAV infected and uninfected HLTE from the same donor (a patient with indication for lung transplantation due to COPD) 24 h after IAV or mock infection. To correct for differences among individual HLTE pieces, single-cell suspensions from 12 infected and 12 uninfected HLTE pieces were pooled to yield one infected and one uninfected sample for single-cell analysis. After excluding dead cells by FACS, we loaded cells (n = 4500) for a targeted recovery of 3500 cells from each pooled sample at a sequencing depth to obtain 50 k reads per cell. Using cell-specific markers to identify cell clusters allowed identification of 11 cell types (Fig. 6A). These included the expected functional cell types of lower airways (airway epithelial cells, multiciliated cells, endothelial cells) and the major immune cell types: macrophages, monocytes, mast cells, B cells, CD4 + and CD8 + T cells, and NK cells. Mito_high T cells correspond to apoptotic T cells with high mitochondrial DNA content, indicating cell death, and were excluded from the host cell gene expression analyses. Extensive expression of markers for tissue resident lymphocytes, CD69 and CD103 [40], indicated that most of the captured lymphocytes were indeed tissue resident and not remnants of peripheral blood cells that had not been removed by washing the tissue (Fig. 6B, Figure S7). We then aimed to identify cells containing viral RNAs (Figure S8). Since IAV is a negative sense RNA virus, viral mRNA is transcribed from the negative sense (−) strand, and identification of a viral transcript (+ strand) therefore reflects viral transcription. However, by scRNAseq it is not easily possible to distinguish between intracellular viral mRNA synthesis in infected cells and uptake or binding of free viral mRNA from the extracellular environment. Of the eight viral genome segments, RNA corresponding to the segment encoding M1 and M2 proteins could not be detected for unknown reasons. Of the remaining seven segments, NS and HA mRNAs were the most abundant, which is consistent with the viral life cycle. Strongest viral mRNA expression per infected cell was seen in epithelial cells, the main host cell supporting productive IAV infection, followed by macrophages, which are known to support a nonproductive IAV infection and contribute to antiviral immune responses. However, there was clear evidence of viral transcripts also in association with the other cell types, whereby levels were lowest in endothelial cells, mast cells, and monocytes. Comparing DE between the same cell types in single cell suspensions from infected and uninfected HLTE enabled us to assess transcriptomic responses in the 10 cell types (Fig. 6C, Figure S9). CD4 T cells (72 DE genes), CD8 T cells (43 DE genes), macrophages (42 DE genes), NK cells (26 DE genes), and mast cells (25 DE genes) mounted the briskest transcriptional responses, whereas only weak responses were seen in multiciliated cells (0), B cells (1), endothelial cells (2), epithelial cells (1), and monocytes (1). Thus, the most robust antiviral host responses did not originate from the main target cells supporting viral transcription (epithelial cells) but from other cell types with less or no evidence of viral mRNA.

**Fig. 7:**
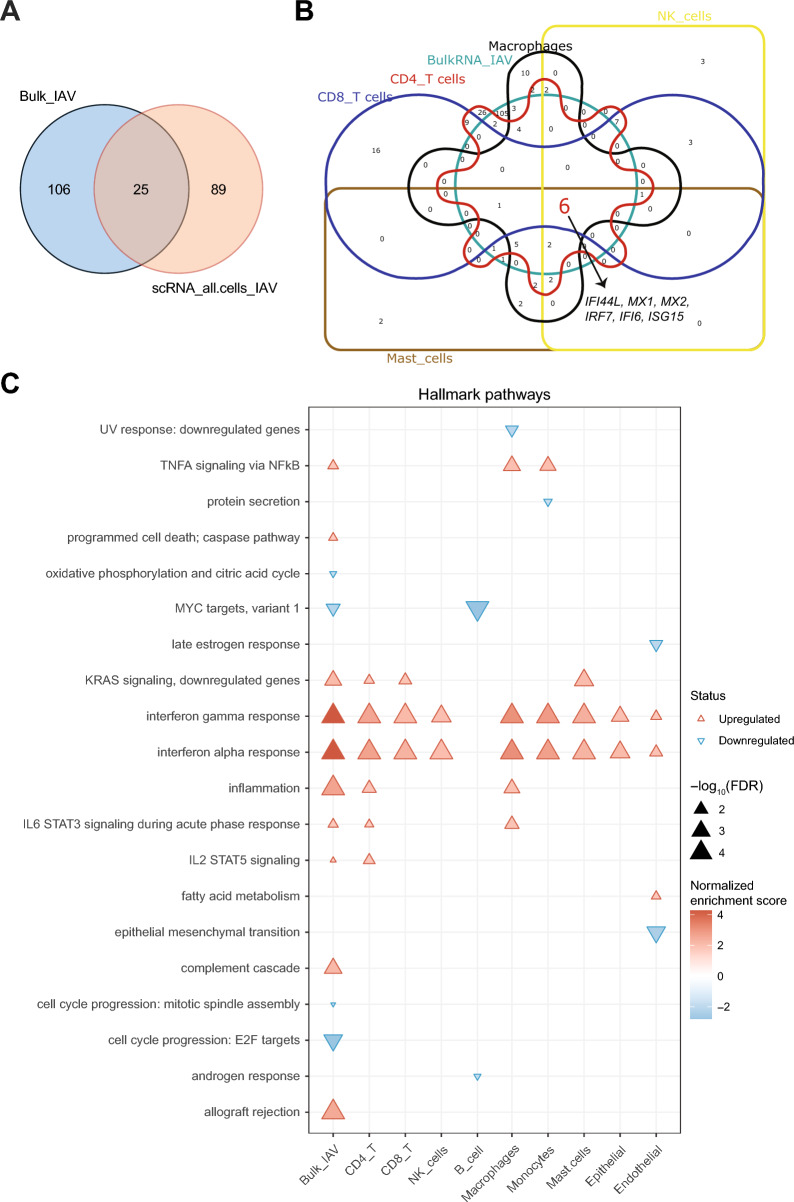
Comparison of DE Genes and functional pathways in IAV-infected HLTEs detected at the tissue or the single cell level 9. **A**, **B** Venn diagrams illustrating the relationships between DE genes identified by bulk RNAseq and by scRNAseq in different cell types. **A** Bulk RNAseq vs. scRNAseq (pool of all cell types); **B** bulk RNAseq vs. scRNAseq (individual cell types indicated in the diagram). The arrow points to 6 mRNAs making up the core tissue response identified by bulk and scRNAseq. **C** GSEA of IAV infection based on the bulk RNAseq data or scRNAseq data. Hallmark pathways [41] are listed on the vertical axis in reverse alphabetical order (top to bottom). Significantly (FDR ≤ 0.05) enriched or depleted pathways are marked with triangles as indicated in the legend on the right

A comparison of DE genes identified by bulk RNAseq and scRNAseq showed that scRNAseq identified 19% of the DE genes that were identified by bulk RNAseq, but it also demonstrated that 89 DE genes were revealed by scRNAseq only (Fig. 7A). Of note, six genes (*IFI44L*, *MX1*, *MX2*, *IRF7*, *IFI6*, and *ISG15*) were identified by scRNAseq as DE in all major immune cells as well as by bulk RNAseq and thus constituted the core of the anti-IAV transcriptomic tissue response in this early time window of IAV infection (Fig. 7B).

Thus, this analysis supported the notion that IAV infects the expected cell types in this HLTE model, that antiviral and inflammatory responses are to some extent due to responses in noninfected “bystander” cells, and that scRNAseq may identify DE transcripts that are not detected as differentially expressed by bulk RNAseq.

We then assessed differences among the cell types in terms of functional responses to IAV infection (Fig. 7C). GSEA (Hallmark) revealed that type 1 and 2 IFN responses were induced in all cell types except B cells. CD4 + T cells and macrophages were the most responsive in that the most pathways (six in each cell type) were regulated. Notably, *TNFA signaling via NFKB* was enriched only in macrophages and monocytes. We then compared GSEA in each of the cell types to the GSEA performed at the tissue level (bulk RNAseq). Regulation of hallmark pathways [41] related to interferon response, inflammation, and MYC targets was detected at the tissue and the single-cell level, whereas five pathways were detected only by scRNAseq.

## Discussion

We have characterized transcriptomic changes in ex vivo HLTEs from patients with end-stage emphysema in response to in vitro infection with IAV and two phylogenetically distinct bacterial pathogens during the first 24 h of infection. The validity of this model is supported by the expected cytokine responses and, in the case of IAV, viral RNA replication. Ex vivo infections of human lung tissue are usually performed in histologically normal tissue such as tumor-free margins from tissue resected during lung tumor surgery. However, in centers with a high volume of lung transplantation, explants from patients with terminal pulmonary disease may be available more frequently. This additional source of human lung tissue will, hopefully, enhance the use of HLTEs for ex vivo infections, thus helping to reduce the use of animal models. However, certain limitations should be considered when interpreting results from HLTEs. Firstly, since the tissue is washed and mostly bloodless, there are no intravascularly circulating immune cells. Secondly, depending on the underlying disease leading to the need for lung transplantation, the tissue may already possess an elevated state of inflammation (“pre-inflamed”) and some cell types may not be fully functional, may be underrepresented, or even absent. Nonetheless, scRNAseq demonstrated the presence of all cardinal target cells and the expected cellular responses to IAV infection in the various cell types. Our study revealed substantial intra- and inter-donor variability (e.g., Fig. 1D). While we were able to reduce the impact of intra-donor variability on the bulk RNAseq analyses by pooling RNA from 2 to 4 tissue pieces from the same donor, it was not possible to reduce the impact of inter-donor variability, which likely results from differences in anatomic site provided by the surgeons and pathologists, disease subtype, infection history, current medications, etc. Furthermore, our study is limited by the absence of data from histologically normal lung tissue (e.g., from tumor-free margins from lung tissue resected during tumor surgery), and we could therefore not assess the impact of COPD/emphysema on the observed changes in gene expression. We focused on emphysema because it is a frequent indication for lung transplantation at our center. After an early pilot study, we excluded HLTEs from patients with cystic fibrosis because of overgrowth with pathogenic bacteria including *P. aeruginosa* and we excluded HLTEs from patients with pulmonary fibrosis because they did not support IAV RNA replication well (Sohail, Samir & Pessler, unpublished data). Further studies should explore the usefulness of HLTEs from other disease indications for lung transplantation such as pulmonary hypertension, sarcoidosis, and others.

Overall, our data suggest that this model is not optimal for identifying infection-associated sncRNAs, at least for IAV and BCG infection. Nonetheless, DE of pathophysiologically plausible miRNA could be identified in *P. aeruginosa* infection. Of note, we also found that expression of the lesser-known sncRNA classes (notably piRNA) changed substantially during *P. aeruginosa* infection. Bulk RNAseq of both coding and noncoding long RNAs revealed strong transcriptomic “footprints” which differed substantially between IAV and the bacterial infections. As expected from an acute *P. aeruginosa* infection, transcriptomic changes (with respect to uninfected tissue) were substantially more pronounced than in BCG infection. However, only very few genes were DE in a two–group comparison between *P. aeruginosa* and BCG, suggesting that the interactions of these two distinct pathogens with host tissue ex vivo in the first 24 h of infection share cardinal features and differ mostly in terms of intensity of the interaction. *P. aeruginosa* more commonly causes colonization or subacute disease, and BCG only causes clinical disease in immunocompromised individuals. Thus, pathogens that cause acute lower airway infections, e.g. pneumococci, would likely lead to even more pronounced transcriptome changes in this HLTE model. We validated several genes that had been implicated in host responses to bacterial infections in other models, notably *GJB2, VNN1,* and *DUSP4.* These results underscore the value of this HLTE model for the validation of genes that were identified in the context of other pathogens, species or model types such as cellular models.

The scRNAseq analysis identified cells with low levels or no IAV mRNA as important sources of IFN responses. This agrees well with previous reports of inflammatory responses originating from such bystander cells. For instance, in a study of IAV infection of human PBMCs we found that viral transcripts were only associated with monocytes, but interferon responses also with T cells, B cells, and NK cells [18]. We found weak expression of IAV transcripts in mast cells. IAV infection of mast cells is of considerable clinical interest, as this virus can trigger hypersensitivity reactions such as asthma and COPD exacerbation in susceptible individuals [42, 43]. Our results suggest that only a small percentage of mast cells harbored IAV mRNA. Further research is, therefore, required to determine the role of direct infection of mast cells to these hypersensitivity reactions. Substantial gene expression changes due to IAV infection were detected in CD4 + cells (Fig. 6B). Considering that blood had been removed from the HLTEs, and the extensive expression of CD69 and CD103, these cells likely represent tissue-resident cells. It is, therefore, possible that these cells mounted a tissue memory response due to prior IAV infection or seasonal influenza vaccination. The identification of the 6-gene ”IAV signature” is particularly intriguing. It demonstrates that this HLTE model recapitulates the induction of genes which have been extensively validated as key players in the cellular response to influenza viruses, and it also shows that these genes are induced across a diverse range of immune cells found in lung. The sequences encoding the three lncRNAs in this signature are all located adjacent to protein-coding genes involved in inflammation and host responses to influenza viruses and are likely co-transcribed with these genes. For instance, *lnc-DDX60-1* is located downstream of the *DDX60* gene, which encodes an RNA binding protein involved in antiviral responses [44], and *RP11-202G18.1* is located upstream of *LPAR1*, which encodes a G-protein coupled receptor involved in pulmonary fibrosis and response to tissue damage and infectious agents [45]. Thus, they may mostly reflect induction of antiviral responses in lung tissue but it is unclear whether they play any functional role. There is a great clinical need for biomarkers that can distinguish between viral and bacterial infections early in the infection in order to start the correct antibacterial or antiviral treatment as early as possible. Further research is necessary to test whether expression of these RNAs can be measured in easier-to-obtain biosamples such as peripheral blood, sputum, or bronchoalveolar lavage and to test whether they are consistently more highly expressed in viral than bacterial infections.

Regulation of cell cycle was a recurring theme in the GSEAs, with a preponderance of cell cycle inhibition. It is well known that IAV infection favors a G0/G1-phase cell cycle arrest in infected cells in order to channel cell resources into production of progeny virions [46]. Further research is required to assess the functional implications of the observed cell cycle arrest in the bacterially infected HLTEs.

In conclusion, our results demonstrate the usefulness of “upcycling” explanted lung tissue from patients with terminal emphysema to study transcriptomic tissue responses in the early phase of viral and bacterial infections. Further research should assess the usefulness of tissue explants from patients with other underlying diagnoses, the validity of the model for infections with other pathogens, and the functional significance of the novel RNA tissue biomarkers identified herein, such as the noncoding RNAs induced by IAV infection.

## Methods and materials

*Preparation and maintenance of Human Lung Tissue Explants (HLTEs).* Bronchial lung tissue explanted for clinical reasons from 22 patients with emphysema due to terminal COPD was obtained from the Institute of Pathology, Hannover Medical School. Demographic information was available for 20 donors: 10 women, 10 men, median age 54.5 years (min–max = 46–64). Further sociodemographic or medical information was not transferred with the explants due to data privacy regulations. The tissue was further dissected into small pieces with an average size of approx. 27 mm^3^ (3 × 3 × 3 mm) and an average weight of approx. 30 mg. These pieces (HLTEs) were cultured in RPMI medium without any supplement. Tissue pieces were cultured overnight in a humidified tissue culture incubator at 37 °C, 5% CO_2_; this step was termed overnight washing. After 12–16 h of washing, tissue pieces were infected with the respective pathogen. This protocol was developed by combining already published methods [8, 18].

*Infections.* Influenza virus (A/Giessen/6/2009 H1N1; abbreviated IAV) [47]. Infection medium containing 2.0 × 10^5^ ffu/ml in RPMI medium was added to a 24 well plate, placing one HLTE piece per well. Plates were incubated at 37 °C for the durations indicated in the figure legends. *Mycobacterium bovis Bacillus Calmette–Guérin (BCG)* was grown under agitation to mid-log phase in 50 ml of Middlebrook 7H9 (Difco) liquid culture medium supplemented with 0.5% glycerol, 0.15% Tween-80 and 10% oleic acid-albumin-dextrose-catalase (BD Biosciences). Bacterial cells were collected in a 50 ml Falcon tube and then washed twice with 45 ml PBS (Gibco) by centrifugation at 4000*G*. Bacterial optical density was measured with a spectrophotometer at an absorbance of 600 nm. Infection medium containing 5x10^6^ CFU/ml was prepared and HLTEs were subjected to infection in a 24 well plate. *P. aeruginosa* strain PA14 was cultured in Luria–Bertani broth at 37 °C in a shaker incubator and harvested at log phase. CFU were estimated according to the OD value measured as described by Kim et al. [48], and infection medium was prepared containing 1 × 10^8^ CFU/ml in RPMI medium. HLTEs were incubated in 24 well plates for 2 h at 37 °C. After infection, HLTEs were washed and incubated in RPMI medium containing 50 μg/ml gentamycin in order to arrest growth of extracellular bacteria.

*Focus forming assay.* Cell culture supernatants containing IAV were serially diluted in PBS supplemented with 0.2% BSA and 1% Ca/Mg solution. Recently confluent monolayers of MDCK cells in 96-well plates were rinsed with PBS and each well infected with 50 μl of tenfold serially diluted virus for 1 h at room temperature. After infection, the inoculum was aspirated and 150 μl Avicel-media (1% Avicel in MEM media supplemented with 0.5% BSA, 2 μg/ml trypsin and 1% dextran) was added to the cell monolayer. The plates were then incubated at 37 °C, 5% CO_2_ for 24 h, followed by removal of the Avicel media and fixation of cells with 4% PFA and 1% Triton X-100. Cells were stained by mouse-anti-NP-antibody followed by anti-mouse-HRP-antibody. AEC-staining-solution was used to visualize IAV positive cells [49].

*LDH release assay.* Supernatants and tissue were collected from HLTE cultures at 0, 24, 48 and 72 h post incubation and stored at 4 °C until collection of the 72 h time point. Tissue was lysed in 1% Triton X-100 lysis buffer, tissue lysate and supernatant were diluted and added to 96-well microplates. Catalyst and dye solution were mixed and added to samples according to the protocol of the Roche LDH cytotoxicity detection kit. Color development was measured by OD at 492 nm (background control at 630 nm). LDH release as percentage was calculated as follows: (100/LDH of lysate) x LDH of sample.

*ELISA.* Supernatants collected from HLTE cultures were immediately stored at − 20 °C. Samples were thawed at 4 °C before measuring concentrations by commercial kits for the following proteins; IP10, IL-1β, IL-6, IL-10, IFN-γ, PROK2. In brief, ELISA plates were coated with specific capture antibodies overnight at 4 °C, followed by washing and blocking with 3% BSA (Roth, 8076.5). Plates were washed and then incubated with standard proteins or sample supernatants (diluted in 1% BSA) for 2 h. After binding of protein antigens to coated antibodies, detection antibodies were added, followed by adding avidin conjugated with HRP. This was followed by a final quadruple wash step, after which the chromomeric substrate 3,3′5,5′-tetramethylbenzidine (TMB) was added and incubated in the dark. After approximately 15 min, the reaction was stopped by adding 0.3 M sulfuric acid (H_2_SO_4_) solution. Plates were read on an ELISA reader (BioTeK, Synergy 2.0) at OD 450 nm and background OD that was earlier measured at 570 was subtracted. Protein concentrations were calculated by comparison with standard curves.

*Comparison of two tissue preservation methods before RNA extraction.* To identify the best HLTE preservation method for RNA extraction, HLTE samples were either stored in RNAlater for 24 h at 4 °C or snap-frozen in liquid nitrogen. Tissues from RNAlater were transferred to −80 °C after 24 h, whereas snap-frozen tissues were immediately transferred to −80 °C until RNA extraction.

*RNA extraction* of HLTEs was performed using miRNeasy Qiagen Kit (Qiagen, 74104) according to the manufacturer’s instructions with a few modifications. Briefly, Qiazol lysis reagent was added directly to frozen tissue, and HLTEs were disrupted and homogenized using an Ultra-Turrax® homogenizer (T10, IKA, 0003737000) for 20–30 s. at the highest speed. Chloroform was added and the resulting mixture was centrifuged 12,000 × *g* for 15 min at 4 °C. The colorless upper layer was transferred to RNA binding columns with 100% ethanol. After two washings of RNA with RPE buffer, DNA was digested by treatment with DNAse I. RNA was eluted into 50 μl RNAse free water. The resulting sample contains total RNA including small RNA (< 50 nt) and therefore can be used for both mRNA and sncRNA profiling.

*RNA quality control.* The RNA quality and quantity were determined using the NanoDrop spectrophotometer (Thermo Fisher Scientific). A 260/280 ratio between 2.0 and 2.2 indicated RNA without contamination. The RNA Integrity Number (RIN) was determined with an Agilent Bioanalyzer using Eukaryote Total RNA Nano Series II chips (Agilent, 5067-1511).

*Complementary DNA (cDNA) generation.* Following RNA extraction, cDNA was prepared using the RT PrimeScript™ Master Mix (Takara) following the manufacturer’s instructions. Briefly, 400 ng of RNA was reverse transcribed by adding 2 µl master mix (5x) and making a total reaction volume of 10 µl, followed by incubation at 37 °C for 15 min. Afterward, all samples were heated to 85 °C for 10 s to inactivate the enzymes. The cDNA thus synthesized was diluted further in RNase free water to get a final RNA concentration of 5 ng/µl of the cDNA.

*Gene expression analysis using real-time PCR.* Primers (Table 1) were designed using either PrimerBLAST (National Center for Biotechnology Information, National Institutes of Health) or the Primer3 online tool [50]. Primers were designed to span exon–intron boundaries, have annealing temperatures around 60 °C and generate amplicons of 100 bp–300 bp. RT-qPCR reactions were set up in a final volume of 20 µl, using the SensiFast™ SYBR® No-ROX Kit (Bioline, Taunton, MA) and the primers listed. RT-qPCR was performed in a LightCycler® 2.0 instrument (Roche, Mannheim, Germany), using 45 cycles of the following program: 95 °C for 15 s., 60 °C for 15 s., and 72 °C for 15 s. To exclude artifacts resulting from primer dimer formation, melting curve analysis was performed using the sequence 95 °C for 15 s., 60 °C for 15 s., 95 °C for 1 min. and 37 °C for 30 s. Relative expression of the mRNA targets was calculated using the 2^−ΔΔCT^ method [51]. Amplification of a single amplicon was confirmed by obtaining dissociation curve (melting curve) profiles as well as by gel electrophoresis to verify the size of the reaction product.

**Table 1:** List of PCR primers used

Gene name	Primer name	Sequence (5′-3′)
*IL-6*	IL6-F	CTACATTTGCCGAAGAGCCC
	IL6-R	CCCTGACCCAACCACAAATG
*HPRT*	HPRT-F	GAACGTCTTGCTCGAGATGTG
	HPRT-R	CCAGCAGGTCAGCAAAGAATT
*PROK2*	PROK2-F	TGACAAGGACTCCCAATGTG
	PROK2-R	TACGAGTCAGTGGATGGCAG
*IL1B*	IL-1β-F	TACCCAAAGAAGAAGATGGAA
	IL-1β-R	GAGGTGCTGATGTACCAGTTG
*CXCL10*	CxCL10_F	CTGCTTTGGGGTTTATCAGA
	CxCL10_R	CCACTGAAAGAATTTGGGC
*IL10*	IL10-F	TACCTGGGTTGCCAAGCCT
	IL10-R	AGAAATCGATGACAGCGCC
*HA*	HA-F	CTCGTGCTATGGGGCATTCA
	HA-R	TTGCAATCGTGGACTGGTGT
RP11-202G18*.1* (ENSG00000227531)	AL414.1-F	ACCAGTGCACCTCATTCAGA
	AL414.1-R	TCCTCCATGCCTTTTCCACT
*GBP1P1* (ENSG00000225492)	GBP1P1-F	TGGAACGTGTGAAAGCTGAG
	GBP1P1-R	CAGTTCAGGCTGGACAGACA
*SIMALR* (ENSG00000226004)	LINC02528-F	ACCTACCTACCGAGAGACCT
	LINC02528-R	CACACCACTGACAGAAACGT
*AC093063.1* (ENSG00000268088)	LGALS14-F	CTGAGTGCACTTTGGCCATT
	LGALS14-R	ATCTCTCCACACTTGCACCA
*LGALS17A* (ENSG00000226025)	LGALS17A-F	GCCTTCCATTTCCGAGTGTA
	LGALS17A-R	TGTAATGGTGGTGGCAAAGA

*Small non-coding RNA (sncRNA) sequencing.* Libraries were prepared using the NEBNext® Small RNA Library Prep Set for Illumina and 1 μg of input RNA. The synthesized cDNA was amplified with 12 PCR cycles. Libraries were prepared separately for each biological replicate. Samples were sequenced on an Illumina HiSeq2500 (2 × 50 bp paired-end), generating 50 bp single reads and ≈16 million reads passing filter for each sample. FASTAQ files obtained after small RNA sequencing were used to annotate the transcripts using the OASIS 2.0 web tool [52]. Briefly, FASTAQ files were compressed using the OASIS compressor and then uploaded to the web tool for annotation. The annotation process comprises removing 3' adapters, quality control, mapping to the reference genome (*Homo sapiens—hg38*), and the counting of reads in each sncRNA for each sample. Contaminating viral or bacterial RNA sequences were removed.

*Bulk RNA sequencing of long RNAs (mRNA, lncRNA, lincRNA, pseudogenes).* Quality and integrity of bulk RNA was checked on an Agilent Technologies 2100 Bioanalyzer (Agilent Technologies; Waldbronn, Germany). The RNA sequencing library was generated from 10 ng total RNA using NEBNext® Single Cell/Low Input RNA Library according to the manufacturer´s protocols. The libraries were sequenced on an Illumina NovaSeq 6000, using the NovaSeq 6000 S1 Reagent Kit (100 cycles, paired-end run 2 × 50 bp) with an average of 5 × 10^7^ reads per RNA sample. Large sets of high‐throughput sequencing reads were mapped against the *Homo sapiens* hg38 reference genome via STAR 2.7.3a tool.

*Transcript normalization and differential expression analysis.* The following analyses were done using the R environment and programming code. DESeq2 was used to normalize the counts of each gene to account for differences in sequencing depth and low count variability. The DESeq2 normalization metric is based on the median of ratios method. Following normalization, DESeq2 was run in a pairwise manner, identifying significantly differentially expressed genes with higher levels of expression in the infection group in comparison to the uninfected group. This method provided functionality for a pair-wise comparison (identifying differentially expressed genes between two groups) in the form of a Wald test, and for multiple comparisons (classically used for time-series data) in the form of a likelihood ratio test. The output contained nominal p-values, False Discovery Rate (FDR) or P-adjusted values (correction for multiple tests computed with the Benjamini–Hochberg procedure) and fold change. DESeq2 used an empirical Bayes shrinkage to detect and correct for dispersion and log2-fold change (LFC) estimates. The R software package Visualization of Differential Gene Expression using R (ViDGER) was used to display differential expression outputs uniformly. To present differential expression using DESeq2 output files, volcano plots were generated using the EnhancedVolcano package. Analysis of Variance (ANOVA) was employed to compare the means of all genes across different groups. The top 75 DE genes were selected based on the resulting *p*-values and presented in a heatmap along with adjusted p-values from the pairwise comparisons (DESeq2). Heatmaps and hierarchical clustering were generated using the pheatmap R package, with gene counts scaled by ‘row’. Additionally, a four-way plot was created using ggplot2 to visualize Wald statistic values from the DESeq2 analysis, where BCG Wald statistic values were plotted on the x-axis, and PA infection Wald statistic values on the y-axis. Output from the DESeq2 analysis is found in Tables S1 (sncRNAs) and S2 (long RNAs).

*Preparation of single-cell suspensions for scRNAseq.* HLTE from one donor was divided into 18 pieces, 9 of which were infected with IAV and 9 subjected to mock infection. After 24 h, the tissue pieces were collected and processed to prepare single-cell suspensions for flow cytometry and cell sorting. Briefly, lung tissues were chopped in digestion buffer (1 mg/ml collagenase D (Roche, 10269638001), 2.0 U/ml Dispase II (Roche, 04942078001) and 0.1 mg/ml DNase I (DNase I, D4527) in Hepes-buffer) with surgical scissors and homogenized with an Ultra-Turrax® dispersing tool (T10, IKA-Werke GmbH, Staufen, Germany) for 40–60 s. The homogenate was incubated for 30 min at 37 °C with mild agitation and then passed through a 30G syringe. The resulting single-cell suspension was filtered through a 40 µm nylon cell strainer and erythrocytes were lysed using ACK (Ammonium-Chloride-Potassium) lysing buffer. Cells were counted using an automated cell counter (Scepter, Millipore, PHCC00000).

*Live/dead cell and apoptosis detection.* Single-cell suspension was washed once with PBS and once with binding buffer. Cells were incubated with fluorochrome-conjugated Annexin V (FITC) for 15 min. After incubation, cells were washed again and resuspended in propidium iodide staining solution. After staining, cells were sorted and analyzed by FACS (Cell Sorter Facility MHH). Dead and duplet cells were excluded from further analysis.

*Single-cell RNA sequencing (scRNAseq).* The 18 samples of sorted live single cells were pooled to generate an IAV-infected and a mock-infected pool. Two single-cell 3’ RNA-Seq libraries were prepared using Chromium Single Cell V3 Reagent Kit and Controller (10 × Genomics) as described in the user guide. In brief, cells (n = 4500) for a targeted recovery of 3500 cells along with gel beads, master mix and partitioning oil were loaded in designated wells on a chromium chip. The chip was placed in a Chromium Controller and run for Gel Bead-in-Emulsion (GEMs) preparation. After completion of the run, GEMs were run in PCR tubes for RT incubation in a thermal cycler. Post GEM-RT incubation, cDNA was cleaned up and amplified. cDNA was washed and quality was evaluated. Gene expression libraries were constructed and then libraries were assessed for quality (TapeStation 4200, Agilent). Libraries were sequenced by NextSeq550 using the NextSeq 500 High Output Kit v2.5 (1 × 75 cycles 400 M cluster). We created a reference package for two species genomes (Human and IAV H1N1) and ran the sequences against this genome package. Initial data processing was performed using the Cell Ranger version 2.0 pipeline (10 × Genomics).

*Single-cell RNA data analysis.* Cell Ranger output files “outs” contained raw counts for each sequenced genes (Human and H1N1 virus) of each cell. These files were used as input for further processing using the R package Seurat 5.0. Following the standard pre-processing workflow, cells were filtered that have unique feature counts over 2500 or less than 200. Raw counts were normalized by the global-scaling normalization method “LogNormalize”. PCA score was used to determine the ‘dimensionality’ of all the cells. Cell clusters were identified as different cell populations based on the expression of their canonical markers recently published. Differential expression was determined for each cell type by comparing the IAV infected cells with uninfected cells. IAV infected cells were identified by the presence of IAV encoded transcripts.

*Gene Set Enrichment Analysis (GSEA).* The gene list and their Wald statistic values from DESeq2 analysis for each pathogen were applied against Hallmark gene set collection databases [41] or Gene Ontology Biological [53] using the online gene enrichment tool WEB-based Gene SeT AnaLysis Toolkit (WebGestalt, www.webgestalt.org) [54]. The R package ggplot2 was used to generate plots displaying gene set terms, their normalized enrichment score (NES), and FDR values.

*Statistics.* Statistical tools used are detailed in the respective text in results or the figure legends. Briefly, GraphPad Prism 8.0.2 (GraphPad Software, Boston, MA) was used to analyze RT-qPCR, ELISA, and LDH release assay results. The Wilcoxon–Mann–Whitney U or unpaired t test were used to assess significance of between-group differences. *P* values were abbreviated as ≤ 0.0001 = ****, ≤ 0.001 = ***, ≤ 0.01 = **, and ≤ 0.05 = *. Data are shown as mean ± SEM. The number of biological replicates is specified in the figure captions as indicated. For transcriptome data, DESeq2 analysis was applied which returned log_2_-fold change, *p*-adjusted values, and *p* values.

## Supplementary Information

Supplementary figures.

Supplementary table 1.

Supplementary table 2.

## Data Availability

The results of the DE analysis with DeSeq2 are provided as Supplemental Tables S1 and S2. The underlying RNAseq datasets are available at GEO https://www.ncbi.nlm.nih.gov/geo/query/acc.cgi?acc = GSE192864, https://www.ncbi.nlm.nih.gov/geo/query/acc.cgi?acc = GSE192528, and https://www.ncbi.nlm.nih.gov/geo/query/acc.cgi?acc = GSE268542.
