# Supplementary material for: Differential transcriptomic host responses in the early phase of viral and bacterial infections in human lung tissue explants ex vivo
Source: Respir Res. 2024 Oct 12;25:369. doi: 10.1186/s12931-024-02988-8 (PMC11471021; doi:10.1186/s12931-024-02988-8)
Supplement: Supplementary file 1 — Supplementary figures. [file 12931_2024_2988_MOESM1_ESM.docx]

**Supplemental files**

Sohail A et al. Differential transcriptomic host responses in the early phase of viral and bacterial infections in human lung tissue explants ex vivo. *Respiratory Research,* 2024.


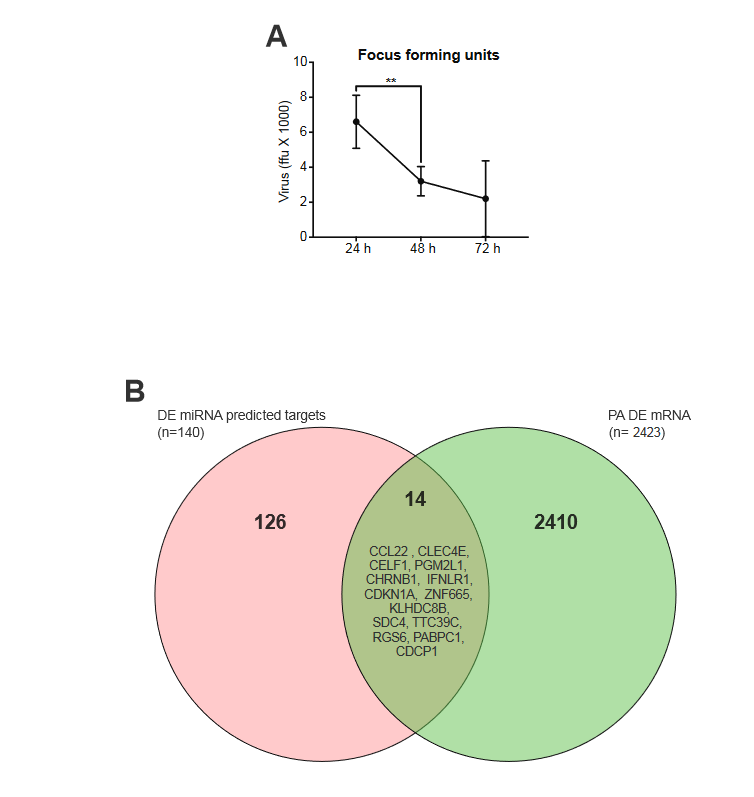


**Figure S1A,** Determination of viable virus particles in tissue culture supernatants within 72 h of infection (foci-forming assay). The initial viral inoculum was not removed, and the curve mostly reflects the declining viability of the inoculum (*n* = 5).

**Figure S1B,** Agreement between predicted targets of DE miRNAs in *P. aeruginosa* infection and DE genes observed by long RNA sequencing. Abbreviation: PA, *P. aeruginosa.*


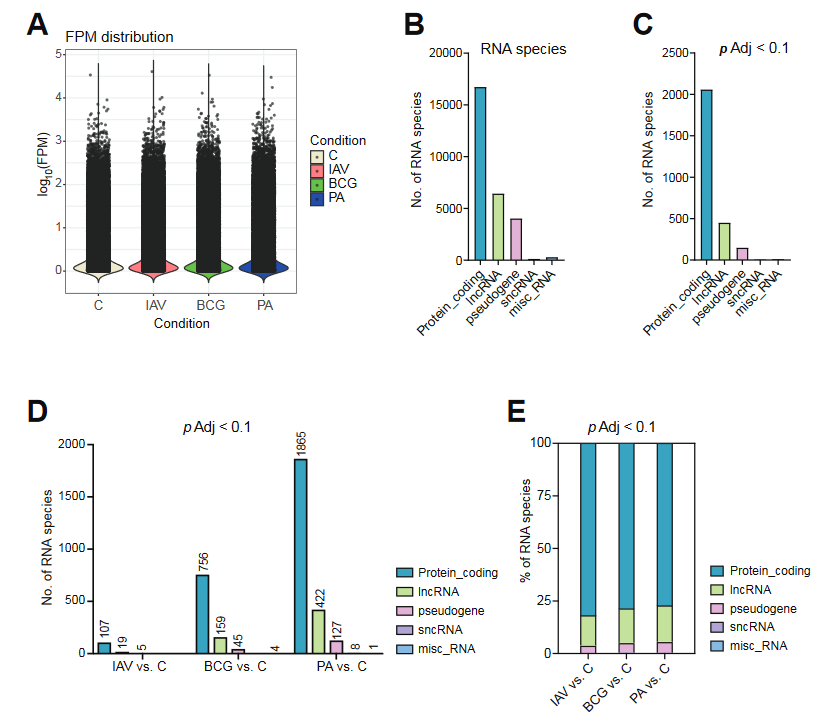


**Figure S2. A**, Fragments per kilobase of transcript per million mapped reads (FPKM) plot indicating equally high RNAseq efficiency across the four experimental groups. **B**, Frequency distribution of detected RNA species corresponding to protein-coding (mRNA), lncRNA, pseudogene transcripts, sncRNA precursors, and misc. RNA. **C,** Frequency distribution of DE (*p-*Adj. ≤0.1) RNA species corresponding to protein-coding (mRNA), lncRNA, pseudogene transcripts, sncRNA precursors, and misc. RNA. **D,E**, Frequency distribution of RNA classes in infection with each of the three pathogens according to number of RNA species (**D**) and relative distribution (%) of each RNA class (**E**). Only polyadenylated lncRNA and pseudogenes are captured by the RNAseq strategy used. The total number of lncRNA and pseudogenes would be higher if polyadenylated species were included. Abbreviations: IAV, influenza A virus; BCG, *Mycobacterium bovis* Bacille Calmette-Guerin; PA, *Pseudomonas aeruginosa.*


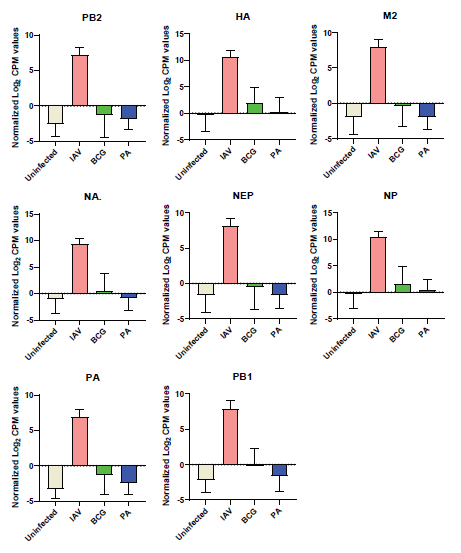


**Figure S3.** Expression of mRNA corresponding to the 8 IAV gene segments in HLTEs, as determined by bulk RNAseq. Data correspond to normalized log2 transformed CPM values.


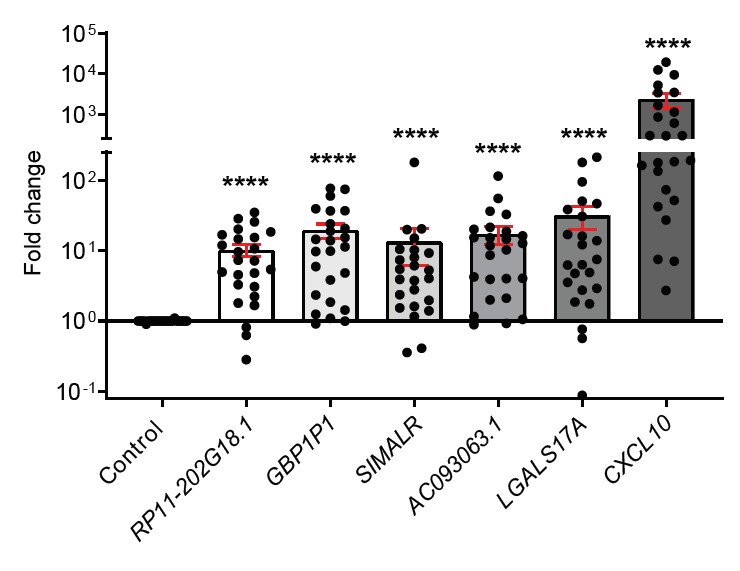


**Figure S4. Induction of five long noncoding RNA species in HLTEs by IAV.** Using independently collected lung explants (n=25 tissue pieces from 14 donors), expression of *RP11-202G18.1*, *GBP1P1*, *SIMALR*, *AC093063.1*, and *LGALS17A* RNA was measured by RT-qPCR by the ΔΔCt method, using *HPRT* mRNA as internal control. Expression in uninfected HLTEs was assigned the reference value of 1. *CXCL10* mRNA expression was measured for comparison. ****, p < 0.0001 (Mann-Whitney U test).


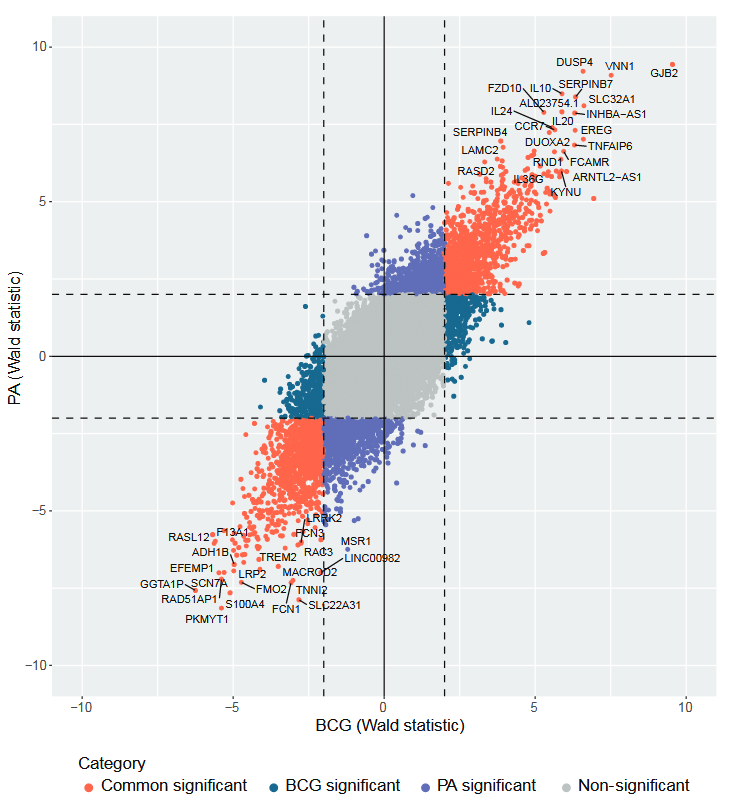


**Figure S5.** Four-way plot illustrating shared and distinct regulation of gene expression by the two bacterial pathogens. Wald statistics for protein-coding RNA, lncRNA, and pseudogene RNA were plotted for PA (y-axis) and BCG infection (x-axis). A Wald statistic of ≥|2| was defined as significant. Positive values indicate upregulation, and negative values downregulation. The plot illustrates that the most significantly differentially expressed RNAs (orange dots) are regulated in the same direction by both pathogens and that the magnitude of significant differential expression was generally higher in *P. aeruginosa* infection. The empty quadrants (upper left and lower right) would contain genes that are regulated in opposite directions.


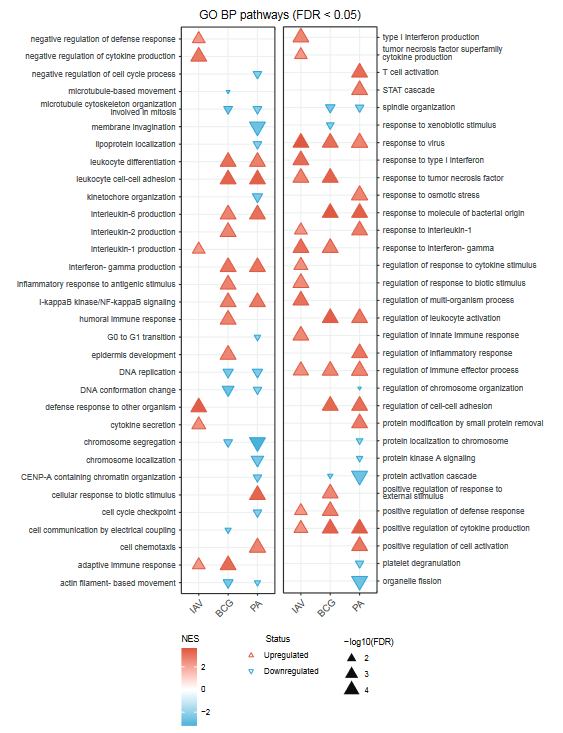


**Figure S6.** Gene set enrichment analysis (Hallmark Biological Pathways) based on bulk RNAseq, contrasting common and distinct biological pathways affected by infection with IAV, BCG, and *P. aeruginosa.* The pathways are arranged in ascending alphabetical order, with a break between “n” and “o”. NES, normalized enrichment ratio.

**CD103 (*ITGAE*)**


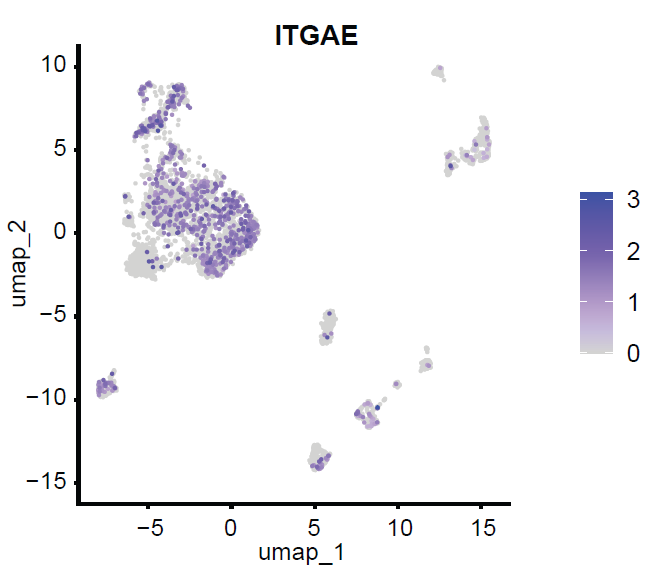


**Figure S7.** Expression of CD103 (integrin alpha-E, encoded by the *ITGAE* gene) in defined cell populations in HLTE, using the scRNAseq dataset visualized in Figure 6A and B. The plot demonstrates extensive expression of this adhesion molecule, which is a marker of tissue resident lymphocytes, in the cluster containing CD4^+^ and CD8^+^ T cells.


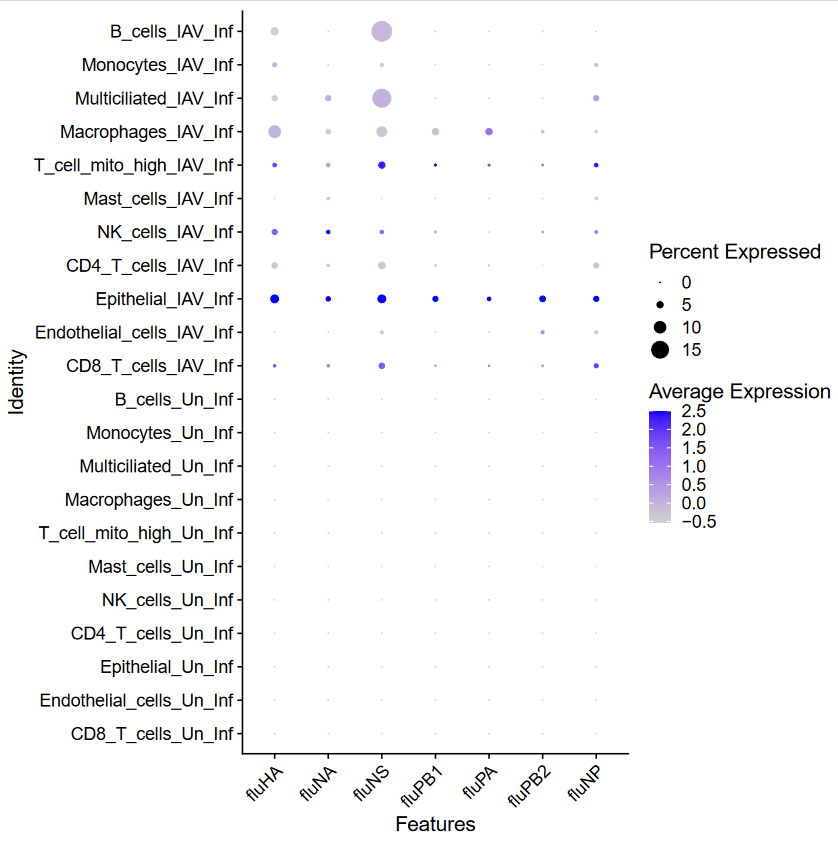


**Figure S8.** **Cell-type specific expression of 7 IAV genome segments.** Mean expression and percentage of cells of the respective cell type that are infected are indicated by color and size of the dots, respectively. The background signal detected in uninfected tissue is shown in the lower half of the panel.


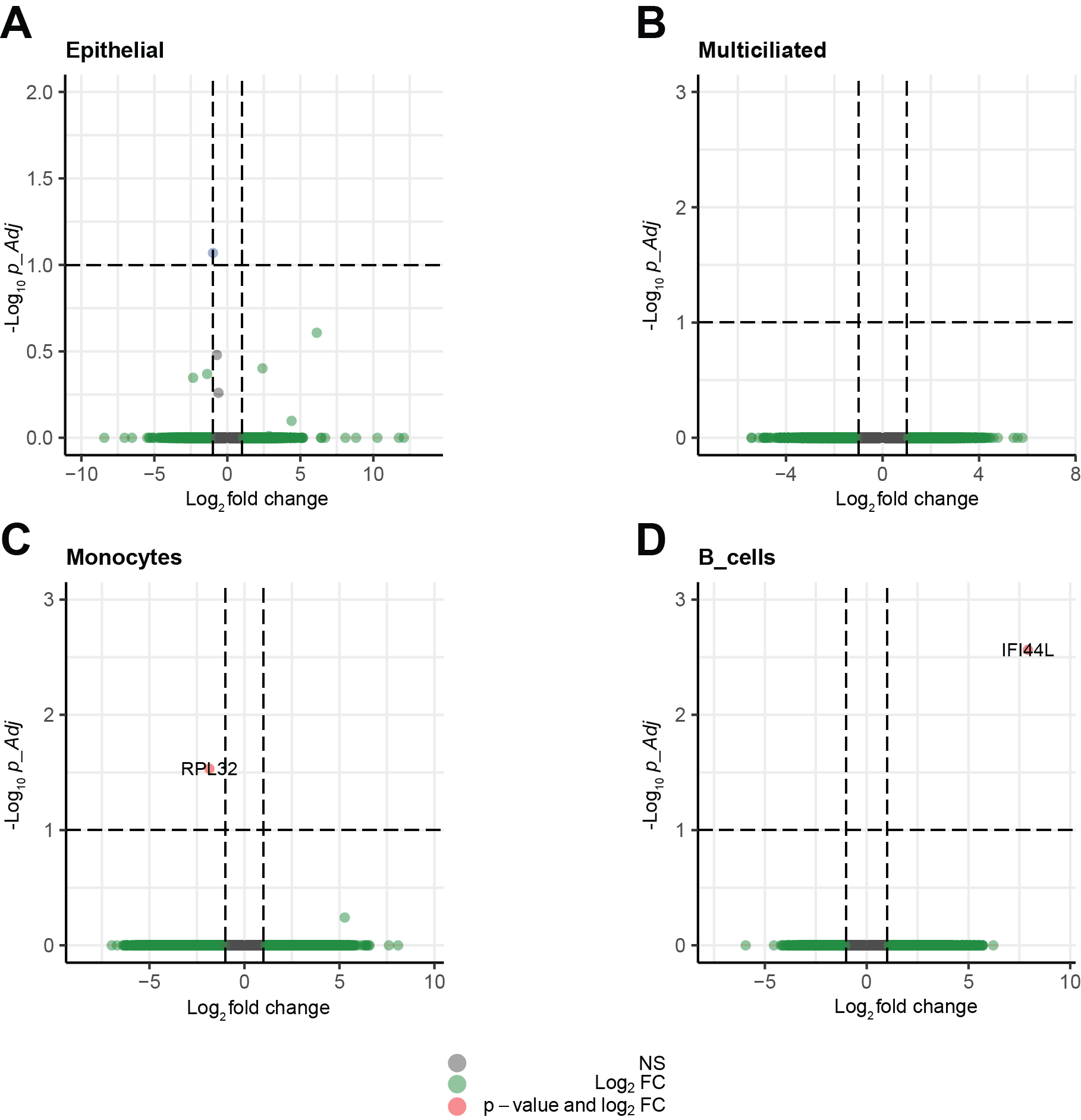


**Figure S9.** Vulcano plots indicating differential expression in scRNAseq analysis: cell types not shown in the main figure (**Figure 6**).
